# Upregulation of Anti-Oxidative Stress Response Improves Metabolic Changes in L-Selectin-Deficient Mice but Does Not Prevent NAFLD Progression or Fecal Microbiota Shifts

**DOI:** 10.3390/ijms22147314

**Published:** 2021-07-07

**Authors:** Sreepradha Eswaran, Anshu Babbar, Hannah K. Drescher, Thomas C. A. Hitch, Thomas Clavel, Moritz Muschaweck, Thomas Ritz, Daniela C. Kroy, Christian Trautwein, Norbert Wagner, Angela Schippers

**Affiliations:** 1 Department of Pediatrics, Faculty of Medicine, RWTH Aachen University, D-52074 Aachen, Germany; seswaran@ukaachen.de (S.E.); Anshu.babbar1@ucalgary.ca (A.B.); mmuschaweck@ukaachen.de (M.M.); 2 Production Animal Health, Faculty of Veterinary Medicine, University of Calgary, Calgary, AB T2N 1N4, Canada; 3 Division of Gastroenterology, Massachusetts General Hospital and Harvard Medical School, Boston, MA 02114, USA; hdrescher@mgh.harvard.edu; 4 Functional Microbiome Research Group, Faculty of Medicine, RWTH Aachen University, D-52074 Aachen, Germany; thitch@ukaachen.de (T.C.A.H.); tclavel@ukaachen.de (T.C.); 5 Institute of Pathology, Ruprecht-Karls-University Heidelberg, D-69117 Heidelberg, Germany; thomas.ritz@med.uni-heidelberg.de; 6 Department of Internal Medicine III, University Hospital, RWTH Aachen, D-52074 Aachen, Germany; danielakroy@gmail.com (D.C.K.); ctrautwein@ukaachen.de (C.T.)

**Keywords:** L-selectin, Nrf2, Keap1, NAFLD, cellular migration, microbiota, oxidative stress, western diet

## Abstract

(1) Background: Non-alcoholic fatty liver disease (NAFLD) is a growing global health problem. NAFLD progression involves a complex interplay of imbalanced inflammatory cell populations and inflammatory signals such as reactive oxygen species and cytokines. These signals can derive from the liver itself but also from adipose tissue or be mediated via changes in the gut microbiome. We analyzed the effects of a simultaneous migration blockade caused by L-selectin-deficiency and an enhancement of the anti-oxidative stress response triggered by hepatocytic Kelch-like ECH-associated protein 1 (Keap1) deletion on NAFLD progression. (2) Methods: L-selectin-deficient mice (Lsel^−/−^Keap1^flx/flx^) and littermates with selective hepatic Keap1 deletion (Lsel^−/−^Keap1^Δhepa^) were compared in a 24-week Western-style diet (WD) model. (3) Results: Lsel^−/−^Keap1^Δhepa^ mice exhibited increased expression of erythroid 2-related factor 2 (Nrf2) target genes in the liver, decreased body weight, reduced epidydimal white adipose tissue with decreased immune cell frequencies, and improved glucose response when compared to their Lsel^−/−^Keap1^flx/flx^ littermates. Although WD feeding caused drastic changes in fecal microbiota profiles with decreased microbial diversity, no genotype-dependent shifts were observed. (4) Conclusions: Upregulation of the anti-oxidative stress response improves metabolic changes in L-selectin-deficient mice but does not prevent NAFLD progression and shifts in the gut microbiota.

## 1. Introduction

Non-alcoholic fatty liver disease (NAFLD) is one of the most prevalent of the chronic and progressive liver diseases, making it the third most common reason for liver transplantations in Western countries [1]. Starting with non-alcoholic fatty liver (NAFL), which is characterized by fat accumulation in the liver (steatosis), it can progress to more severe disease stages such as non-alcoholic steatohepatitis (NASH) with hepatocyte ballooning, inflammation, and/or fibrosis. Finally, it can develop into end-stage disease including liver cirrhosis or hepatocellular carcinoma [2]. The first process in NAFLD development is the accumulation of liver fat, which is considered as an important part of the hepatic component of the metabolic syndrome and associated with insulin resistance [3]. Obesity, mainly caused by the consumption of a Western diet (WD) containing an excess of saturated fats, refined carbohydrates, and fructose-rich beverages, is a key risk factor for the onset of NAFLD [4]. The mechanisms driving progression from simple steatosis to NASH seem to require additional challenges e.g., increased oxidative stress, mitochondrial dysfunction, or aberrant inflammatory immune cell infiltration, which have been implicated as “multiple hits” contributing to liver disease [5]. In addition, liver crosstalk with other organs such as inflamed adipose tissue and the gut can modify immune responses and contributes to progressive pathogenesis. NASH is accompanied by qualitative and quantitative changes in the enteric microbiota and the intestinal barrier that may facilitate the translocation of bacteria/bacterial products into portal circulation, thereby promoting liver inflammation and fibrogenesis [6,7,8]. A detailed description of these mechanistic links, which is a prerequisite for the development of therapies against NAFLD progression, is still lacking.

NAFLD is accompanied by the recruitment of activated inflammatory leukocytes into the liver [9,10]. Immune cell recirculation is a highly regulated and rigorously controlled, multistep process involving different chemokines and cell adhesion molecules [11]. L-selectin (CD62L) is a cell adhesion molecule expressed by many leukocytes, including neutrophils, monocytes, and most types of lymphocytes [12,13]. It mediates the initial attachment and subsequent rolling of leukocytes on activated endothelium, contributing to the pathogenesis of several diseases, including aggravation of inflammation in a liver ischemia reperfusion model [14,15,16,17,18]. Most recently, we have shown that increased L-selectin expression in patients correlates with the progression of NAFLD [19]. Furthermore, the therapeutic blockade or deletion of L-selectin in mice partially protects them from the progression of WD-induced steatohepatitis. WD-treated L-selectin-deficient mice exhibited less fat accumulation and steatosis and fewer metabolic changes with improved glucose tolerance and decreased cholesterol and triglyceride levels when compared to equally treated wild-type (WT) mice. Ameliorated disease was accompanied by increased intrahepatic numbers of anti-inflammatory regulatory T cells (Treg cells), decreased numbers of neutrophils, and activation of the anti-oxidative stress response [19].

Disturbance of the balance between cellular production of reactive oxygen species (ROS) and the capacity to detoxify reactive intermediates is considered a key contributor to NASH progression [20,21]. Elevated ROS concentrations can induce hepatocyte death and lipid peroxidation, which further amplifies liver damage and contributes to hepatic stellate cell activation with subsequent deposition of extracellular matrix [22,23]. Therefore, the expression of antioxidant proteins and detoxifying enzymes is crucial for defense against oxidative stress. Expression of the respective cytoprotective genes is controlled by the promotor regions of antioxidant responsive elements (AREs). These are mainly regulated by the transcription factor erythroid 2-related factor 2 (Nrf2) [24,25,26]. The Nrf2/ARE pathway is modulated by the actin-anchored Kelch-like ECH associated protein 1 (Keap1), which acts as an Nrf2 repressor. In homeostasis, Nrf2 is associated with Keap1 and localized in the cytoplasm. Upon activation by ROS, Keap1 dissociates from Nrf2, which translocates into the nucleus. There, it binds, along with other nuclear proteins, to ARE sequences, triggering the expression of cytoprotective target genes [25]. Nrf2 affects the expression of nearly 500 genes coding for proteins involved in phase II metabolic processes such as NAD(P)H:quinione oxidoreductase 1 (NQO1), glutathion S-transferase (GST), glutathione peroxidase (GSH-Px), ferritin, and heme oxygenase-1 (HO-1). It promotes NADPH production and also induces further antioxidant genes, preventing liver injury. Phase II enzymes detoxify the intermediate metabolites generated by phase I reactions, resulting in a rapid excretion of toxic xenobiotics. In addition, Nrf2 plays an important role in modulating anti-inflammatory responses, autophagy, and proteasome activity [27,28]. Either by deleting Nrf2 or activating Nrf2, e.g., via Keap1 deletion, a number of in vivo mouse studies have addressed the role of Nrf2 in diet-induced obesity. Although some findings are controversial, overall, data from Nrf2 activation show beneficial results [29,30].

Previously, it was shown that hepatocyte-specific deletion of Keap1 in mice reduces acetaminophen toxicity and methionine- and choline-deficient (MCD)- or WD-induced steatohepatitis [31,32,33]. Here, we have studied the effects of simultaneously blocking two NAFLD promoting challenges, namely increased immune cell infiltration and oxidative stress, by analyzing the development of WD-induced NAFLD and fecal microbiota changes in L-selectin-deficient mice with enhanced hepatocytic Nrf2 activation. In terms of body weight, glucose tolerance, and epididymal fat deposition, the enhanced activation of Nrf2 further improved the beneficial outcome of WD-induced NAFLD in L-selectin-deficient mice. Although WD feeding per se induced a significant shift in the fecal microbiota structure, hepatocytic Nrf2 activation appeared to have no major influence in that respect.

## 2. Results

### 2.1. Nrf2 Activation in Hepatocytes of L-Selectin-Deficient Mice Results in Increased Expression of Nrf2 Target Genes

**Figure 1:**
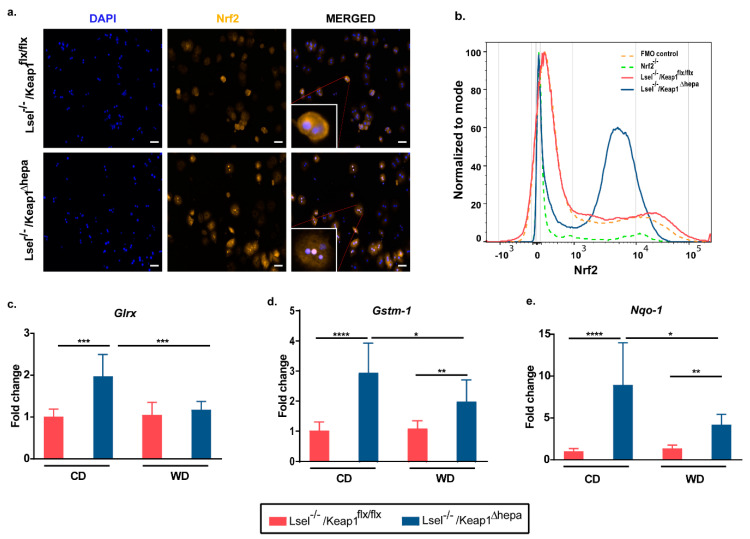
Lsel^−/−^Keap1^Δhepa^ mice exhibit increased Nrf2 expression and an upregulation of Nrf2 target genes in the liver. (**a**) Representative immunofluorescence staining images indicate nuclear translocation of Nrf2 in hepatocytes of Lsel^−/−^Keap1^Δhepa^ mice. Hepatocytes of untreated mice were labeled with anti-Nrf2 (orange) and DAPI (blue). Scale bar: 50 µm. (**b**) Representative flow cytometric analysis indicates increased Nrf2 in hepatocytes of Lsel^−/−^Keap1^Δhepa^ mice. (c-e) Levels of the mRNAs indicated were measured in liver tissue of Lsel^−/−^Keap1^flx/flx^ mice (red bars) and Lsel^−/−^Keap1^Δhepa^ mice (blue bars) after 24 weeks of feeding with chow diet (CD) or Western diet (WD). mRNA levels are expressed as fold increase over the mean value obtained for healthy control liver tissue from Lsel^−/−^Keap1^flx/flx^ *mice.* (Lsel^−/−^Keap1^flx/flx^ CD-fed (*n* = 6), *Lsel^−/−^Keap1^flx/flx^* WD-fed (*n* = 12), Lsel^−/−^Keap1^Δ hepa^ CD-fed (*n* = 4), *Lsel^−/−^Keap1^Δhepa^* WD-fed (*n* = 8). (**c**) *Glrx (glutaredoxin)*, (**d**) *Gstm-1* (*glutathion S-transferase1*) (**e**) *Nqo-1 (NAD(P)H quinone dehydrogenase 1)* Statistical significance was calculated by the one-way ANOVA (analysis of variance). Values are represented as mean ± SD. * *p* ≤ 0.05, ** *p* ≤ 0.01, *** *p* ≤ 0.001, **** *p* ≤ 0.0001.

Recently, we have shown that L-selectin-deficient mice are partially protected from a progression of WD-induced NAFLD [19]. To find out whether an enhanced hepatocytic expression of cytoprotective genes in this system provides an additional benefit in decelerating disease progression, we generated L-selectin-deficient mice with selective hepatic deletion of Keap1 (Lsel^−/−^Keap1^Δhepa^) by crossbreeding L-selectin-deficient mice with Keap1^Δhepa^ mice. This particular Keap1^Δhepa^ mouse strain was reported previously to show a constitutive increase of Nrf2 protein expression in the liver [33,34]), which we verified for the Lsel^−/−^Keap^Δhepa^ mice by flow cytometry and immunofluorescence (Figure 1b). In addition, we observed a trend toward decreased ROS production in hepatocytes of Lsel^−/−^Keap1^Δhepa^ mice when compared to Lsel^−/−^Keap1^flx/flx^ mice. However, this was not significant due to the high variability between the different hepatocyte preparations (Supplementary Figure S1), which was most probably caused by the harsh enzymatic preparation procedure. 

Then, we compared the disease outcome in Lsel^−/−^Keap1^Δhepa^ mice and L-selectin-deficient littermates (Lsel^−/−^Keap1^flx/flx^) after 24 weeks of WD treatment. First, we investigated whether the selective hepatic deletion of Keap1 is reflected by changes in the expression levels of prototypical Nrf2 target genes in liver tissue homogenates. As expected, Nrf2 activation in Lsel^−/−^Keap1^Δhepa^ mice caused a significant increase in the hepatic mRNA expression of the Nrf2 targets glutaredoxin (GLRX), glutathion S-transferase-1 (GSTM-1), and NAD(P)H:quinione oxidoreductase 1 (Nqo1) when compared to Lsel^−/−^Keap1^flx/flx^ mice (Figure 1c–e). Interestingly, WD treatment caused a decrease in the expression of the respective genes in the liver of Lsel^−/−^Keap1^Δhepa^ genes, but in comparison to similarly treated Lsel^−/−^Keap1^flx/flx^ mice, the expression was still significantly elevated. A downregulation of Nrf2 target genes through diet-induced obesity has already been observed in several studies and may be caused by post-translational Nrf2 regulation [35,36,37].

### 2.2. Nrf2 Activation in Hepatocytes of L-Selectin-Deficient Mice Partially Protects Mice from WD-Induced Metabolic Dysfunction 

**Figure 2:**
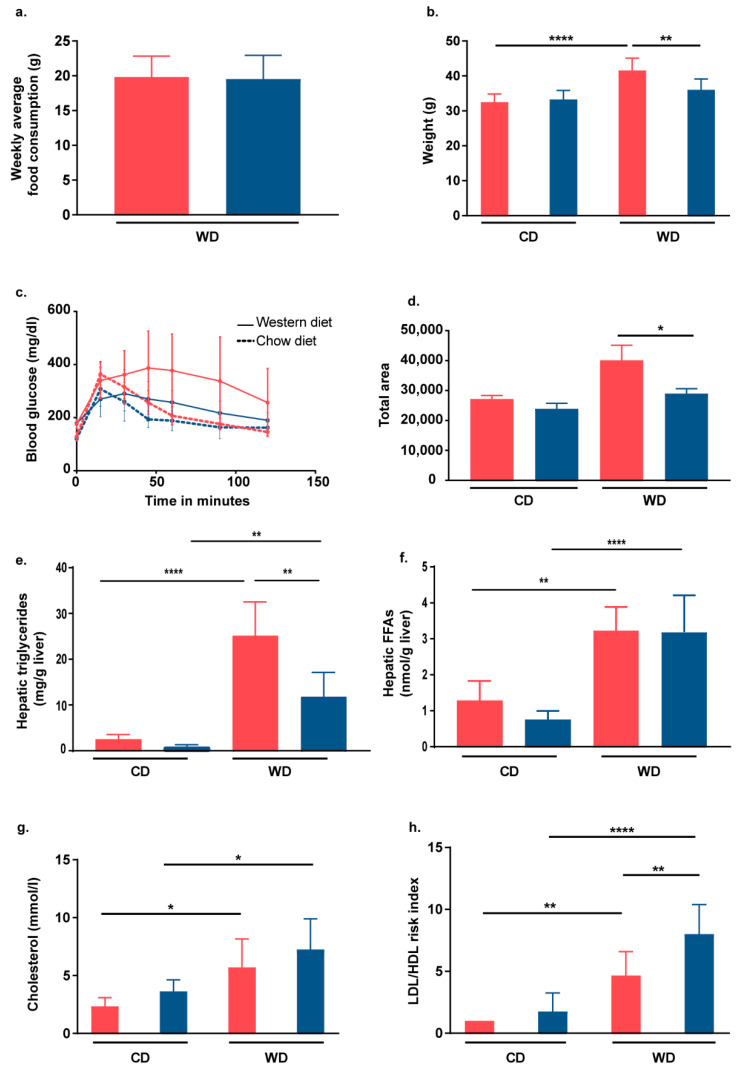
Lsel^−/−^Keap1^Δhepa^ mice exhibit partial protection from metabolic dysfunction. Lsel^−/−^Keap1^flx/flx^ mice (shown in red) and Lsel^−/−^Keap1^Δhepa^ mice (shown in blue) were fed for 24 weeks with chow diet (CD) or Western diet (WD). (**a**) Weekly average WD consumption of Lsel^−/−^Keap1^flx/flx^ *mice* (*n* = 4) and Lsel^−/−^Keap1^Δhepa^ mice (*n* = 4). (**b**) Endpoint body weight, (**c**) endpoint glucose tolerance test (GTT), and (**d**) corresponding quantification as area under curve of Lsel^−/−^Keap1^flx/flx^ CD-fed (*n* = 6), Lsel^−/−^Keap1^flx/flx^ WD-fed (*n* = 12), Lsel^−/−^Keap1^Δhepa^ CD-fed (*n* = 4), and Lsel^−/−^Keap1^Δhepa^ WD-fed mice (*n* = 8). (**e**) Liver triglycerides and (**f**) liver free fatty acids (FFA) of Lsel^−/−^Keap1^flx/flx^ CD-fed (*n* = 5), Lsel^−/−^Keap1^flx/flx^ WD-fed (*n* = 5), Lsel^−/−^Keap1^Δhepa^ CD-fed (*n* = 4), and Lsel^−/−^Keap1^Δhepa^ WD-fed mice (*n* = 5). (**g**) Serum cholesterol and (**h**) LDL/HDL risk of Lsel^−/−^Keap1^flx/flx^ CD-fed (*n* = 6), Lsel^−/−^Keap1^flx/flx^ WD-fed (*n* = 12), Lsel^−/−^Keap1^Δhepa^ CD-fed (*n* = 4), and Lsel^−/−^Keap1^Δhepa^ WD-fed mice (*n* = 8). Statistical significance was calculated either by the one-way ANOVA (**a**,**b**) or unpaired *t*-test (**d**). Values are represented either as mean ± SD (**a**–**c**,**e**–**h**) or mean ± SEM (**d**). * *p* ≤ 0.05, ** *p* ≤ 0.01, **** *p* ≤ 0.0001.

To study the effect of additional Nrf2 activation on obesity, we measured the endpoint body weight of mice that had received either WD for 24 weeks or a normal chow diet. Although the two mouse strains showed the same weekly average food consumption of ≈20 g (Figure 2a), only Lsel^−/−^Keap1^flx/flx^ mice displayed a significant increase in body weight after WD feeding (Figure 2b). This phenotype was also observed in the Keap1^Δhepa^ mouse strain and ascribed to a shift in hepatic metabolism toward increased lipid catabolism and reduced liponeogenesis [33]. Moreover, after 24 weeks of WD feeding, Lsel^−/−^Keap1^Δhepa^ mice exhibited less pronounced features of the metabolic syndrome when compared to similarly treated Lsel^−/−^Keap1^flx/flx^ mice, as demonstrated by faster falls in glucose levels after glucose injection (Figure 2c,d). While serum triglyceride levels were unaffected by either diet or genotype (data not shown), WD treatment caused significantly increased levels of liver triglycerides in both strains, but these levels were significantly reduced in Lsel^−/−^Keap^Δhepa^ mice in comparison to WD-treated Lsel^−/−^Keap^fl/fl^ mice (Figure 2e). With respect to chow-fed controls, non-esterified free fatty free acid levels were significantly elevated in the livers of the treated mice, but did not vary between the different genotypes (Figure 2f).

In addition, serum cholesterol increased in both strains when fed a WD (Figure 2g). When investigated further, the LDL/HDL ratio was found to be significantly increased by WD, with the highest ratio occurring in Lsel^−/−^Keap1^Δhepa^ mice fed WD (Figure 2h). Of note, a mild hypercholesterolemia has already been observed due to Keap1 deletion in WT mice and was attributed to a reduced expression of the Cyp7a1 (cytochrome P450 family 7 subfamily A member 1) gene, which encodes for an enzyme involved in the biotransformation of cholesterol into bile acids [33]. 

### 2.3. Nrf2 Activation in Hepatocytes of L-Selectin-Deficient Mice Has No Major Influence on WD-Induced NASH Progression

**Figure 3:**
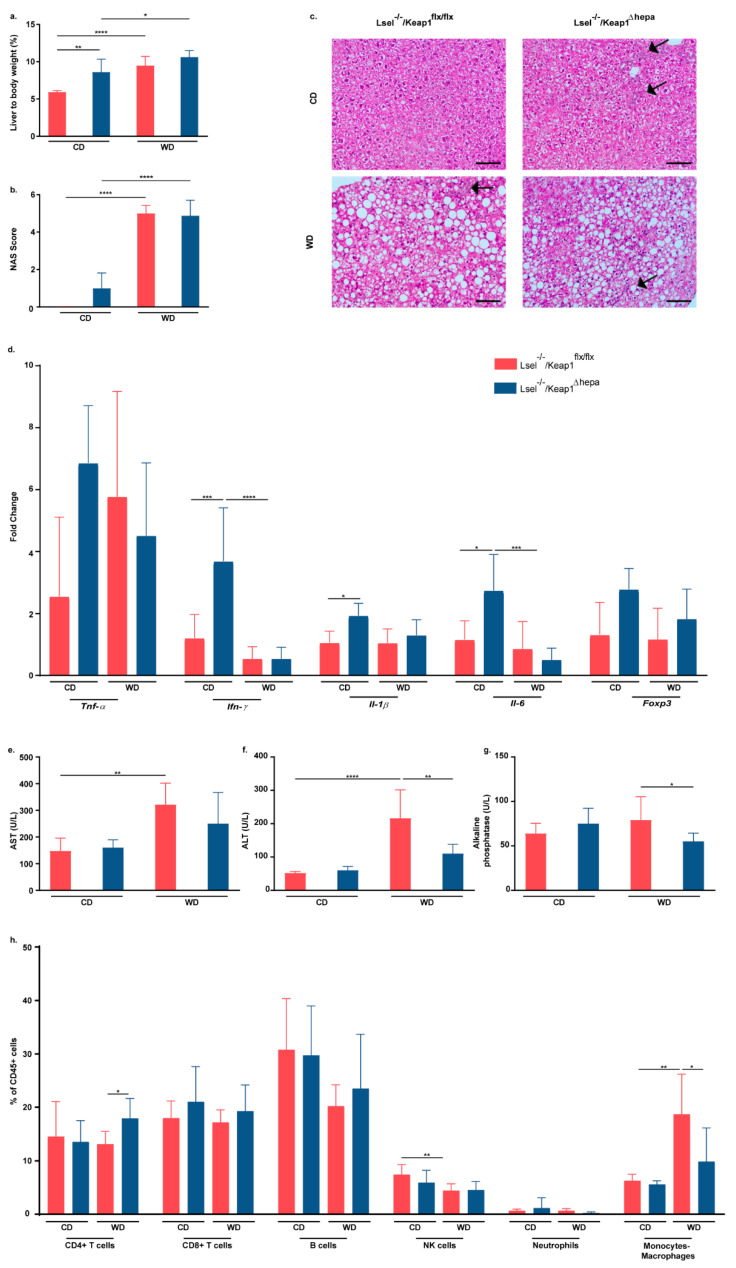
Lsel^−/−^Keap1^Δhepa^ mice are not additionally protected from WD-induced NASH progression. Lsel^−/−^Keap1^flx/flx^ mice (shown in red) and Lsel^−/−^Keap1^Δhepa^ mice (shown in blue) were fed for 24 weeks on chow diet (CD) or Western diet (WD). Shown are the endpoint values of Lsel^−/−^Keap1^flx/flx^ CD-fed (*n* = 6), Lsel^−/−^Keap1^flx/flx^ WD-fed (*n* = 12), Lsel^−/−^Keap1^Δhepa^ CD-fed (*n* = 4), and Lsel^−/−^Keap1^Δ hepa^ WD-fed mice (*n* = 8). (**a**) Liver/body weight ratio. (**b**) NAFLD activity score (NAS). (**c**) Representative images of H&E-stained liver sections of the indicated mice strains. Black arrows indicate the presence of inflammatory foci (original magnification X 20, scale bar = 100 µm). (**d**) mRNA levels of inflammatory mediators in whole liver tissue, expressed as fold increase over the mean value obtained for healthy control liver tissue from Lsel^−/−^Keap1^flx/flx^ mice. (**e**) Quantification of serum aspartate aminotransferase (AST). (**f**) Quantification of serum alanine aminotransferase (ALT). (**g**) Quantification of alkaline phosphatase (ALP). (**h**) Comparative immune cell analysis of liver by flow cytometry. Representative FACS dot plots illustrating the gating strategy are shown in Supplementary Figure S1. Depicted are the percentages of CD45^+^ cells of CD4^+^ T cells (CD45^+^CD3^+^CD8^-^CD4^+^), CD8^+^ T cells (CD45^+^CD3^+^CD4^-^CD8^+^), B cells (CD45^+^CD3^-^CD19^+^), NK cells (CD45^+^NK1.1^+^), neutrophils (CD45^+^CD11b^+^Ly6G^+^), and monocytes/macrophages (CD45^+^Ly6G^-^CD11b^+^F4/80^+^). Statistical significance was calculated by the one-way ANOVA. Values are represented as either as mean ± SD (**a**,**b**,**d**–**h**). * *p* ≤ 0.05, ** *p* ≤ 0.01, *** *p* ≤ 0.001, **** *p* ≤ 0.0001.

Under conditions of chow feeding, Lsel^−/−^Keap1^Δhepa^ mice displayed an increased liver/body weight ratio when compared to Lsel^−/−^Keap1^flx/flx^ mice. This was not unexpected, because an increased liver/body weight ratio has already been described in mice with hepatocytic Keap1 deletion [33]. After 24 weeks of WD feeding, liver/body weight ratios in both mouse strains were significantly elevated with respect to their chow-fed controls, but they did not differ significantly between Lsel^−/−^Keap1^flx/flx^ and Lsel^−/−^Keap1^Δhepa^ mice (Figure 3a). Histological analysis of the liver using H&E staining revealed a significant increase in hepatocyte ballooning with micro- and macrosteatosis in both mouse strains after 24 weeks of WD but no difference between Lsel^−/−^Keap1^flx/flx^ and Lsel^−/−^Keap1^Δhepa^ mice (Figure 3b,c). Lsel^−/−^Keap1^Δhepa^ mice had a slightly increased NAFLD activity score (NAS) under conditions of chow diet, which was not significant when compared to similarly fed Lsel^−/−^Keap1^flx/flx^ mice. We also analyzed the expression levels of genes that are known to change during NAFLD progression via RT-PCR from liver tissue homogenates. In addition to a small decrease in interleukin-6 (IL-6) mRNA and a trend toward increased Forkhead box P3 (FoxP3) levels in Lsel^−/−^Keap1^Δhepa^ mice, which was not significant, WD feeding did not cause a change in inflammatory gene expression in the livers of either mouse strain. However, under basal conditions of chow feeding, Lsel^−/−^Keap1^Δhepa^ mice did show an increase in gene expression in the pro-inflammatory mediators interferon-γ (Ifn-γ), Il-1β, and Il-6 (Figure 3d). Although we did not observe any difference in NAS scores between WD-treated Lsel^−/−^Keap1^flx/flx^ and Lsel^−/−^Keap1^Δhepa^ mice, the plasma levels of both alanine aminotransferase (ALT) and aspartate aminotransferase (AST) were found to be significantly increased only in Lsel^−/−^Keap1^flx/flx^ mice. This may suggest that Nrf2 activation protects Lsel^−/−^Keap1^Δhepa^ mice to some extent from liver damage (Figure 3e,f). In line with these results, after 24 weeks of WD treatment, Lsel^−/−^Keap1^Δhepa^ mice exhibited significantly decreased levels of alkaline phosphatase (ALP) when compared to their similarly treated Lsel^−/−^Keap1^flx/flx^ littermates (Figure 3g). Increased intrahepatic inflammatory immune cell immigration is a characteristic feature of progressing NAFLD. Therefore, we performed flow cytometric analysis of different liver immune cell populations. Detailed analysis of major leukocyte groups included CD4^+^ T cells, CD8^+^ T cells, B cells, NK cells, neutrophils, and monocytes/macrophages. Under chow diet conditions, we could not find any striking alterations in the frequencies of these cell populations caused by the Nrf2 activation. As expected, we observed only very low frequencies of neutrophils, which can be attributed to the L-selectin-deficiency, in both mouse strains [19]. WD feeding resulted in increased CD4^+^ T cell frequencies in the livers of Lsel^−/−^Keap1^Δhepa^ mice and decreased CD11b^+^F4/80^+^ monocyte/macrophage frequencies when compared to Lsel^−/−^Keap1^flx/flx^ littermates (Figure 3h). Whereas CD4^+^ T cells are mainly thought to provide protection from WD-induced pathologies [38], monocytes/macrophages seem to be central players in the progression of NAFLD [39].

### 2.4. Nrf2 Activation in Hepatocytes of L-Selectin-Deficient Mice Results in a Decreased Epidydimal White Adipose Tissue/Body Weight Ratio

**Figure 4:**
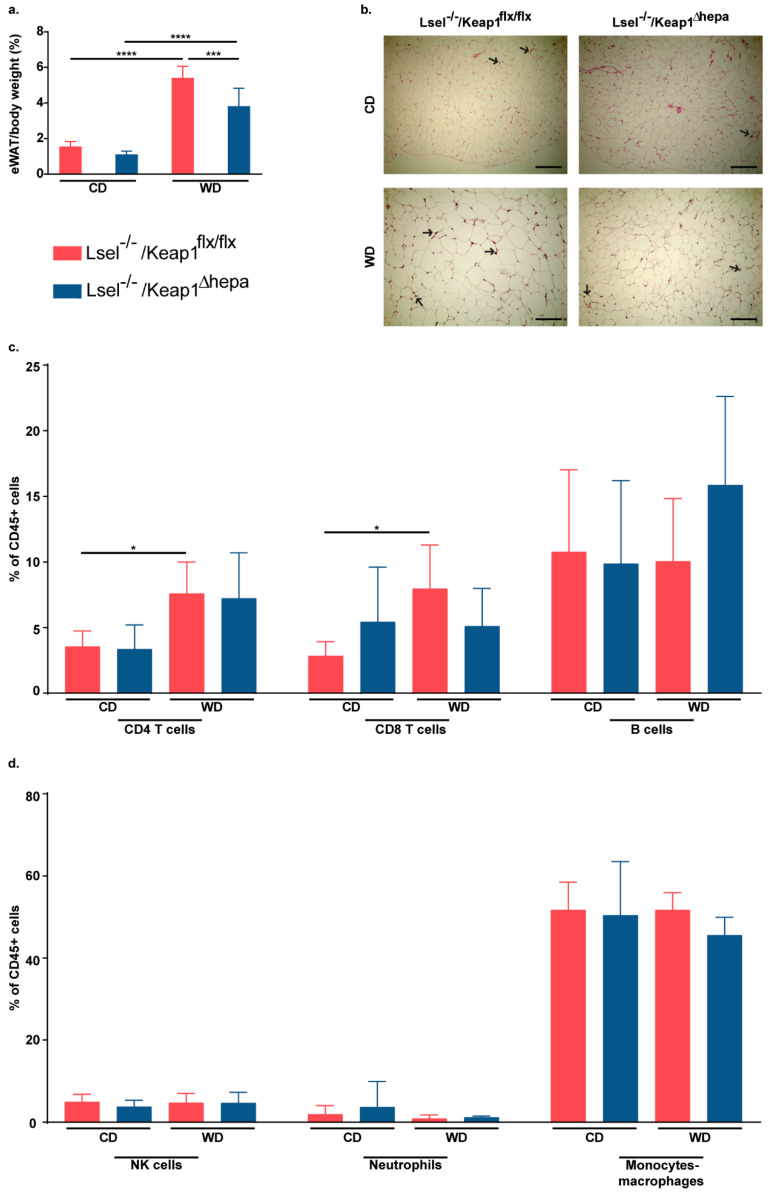
Epidydimal white adipose tissue of WD-fed Lsel^−/−^Keap^∆hepa^ mice exhibit decreased immune cell infiltration. Lsel^−/−^Keap1^flx/flx^ mice (shown in red) and Lsel^−/−^Keap1^Δhepa^ mice (shown in blue) were fed for 24 weeks on chow diet (CD) or Western diet (WD). Depicted are the endpoint values of Lsel^−/−^Keap1^flx/flx^ CD-fed (*n* = 6), Lsel^−/−^Keap1^flx/flx^ WD-fed (*n* = 12), Lsel^−/−^Keap1^∆hepa^ CD-fed (*n* = 4), and Lsel^−/−^Keap1^Δhepa^ WD-fed mice (*n* = 8). (**a**) Epidydimal white adipose tissue (eWAT)/body weight ratio. (**b**) Representative images of H&E stained eWAT sections of the indicated mice strains (original magnification X 20, scale bar = 100 µm. Areas with immune cell infiltration are labeled with black arrows. (**c**,**d**) Comparative immune cell analysis of eWAT by flow cytometry. Representative FACS dot plots illustrating the gating strategy are shown in Supplementary Figure S2. Depicted are the percentages of CD45^+^ cells of (**c**) CD4^+^ T cells (CD45^+^CD3^+^CD8^-^CD4^+^), CD8^+^ T cells (CD45^+^CD3^+^CD4^-^CD8^+^), B cells (CD45^+^CD3^-^CD19^+^), and (**d**) NK cells (CD45^+^NK1.1^+^), neutrophils (CD45^+^CD11b^+^Ly6G^+^), and monocytes/macrophages (CD45^+^Ly6G^-^CD11b^+^F4/80^+^). Statistical significance was calculated by the one-way ANOVA. Values are represented as either as mean ± SD (**a**,**c**,**d**). * *p* ≤ 0.05, *** *p* ≤ 0.001, **** *p* ≤ 0.0001.

NAFLD is strongly associated with obesity, and inflamed adipose tissue seems to promote the progression to NASH [40].Hepatocytes comprise up to 80% of the total liver cell population and fulfill key functions. In addition to filtering of blood, nutrient uptake, and detoxification of substances such as alcohol, their functions include the secretion of proteins and lipids and the formation of bile [41]. Therefore, the disturbance of hepatocyte functions, e.g., by increased oxidative stress, could potentially affect all of these processes and have an influence on adipose tissue inflammation, which is accompanied by increased immune cell infiltration, and gut microbiota. Thus, we next analyzed the epidydimal white adipose tissue (eWAT), which is one of the main fat deposits of the body. In both mouse strains, WD feeding caused an increased eWAT/body weight ratio when compared to the respective chow fed controls. However, this increase was significantly lower in the Lsel^−/−^Keap1^Δhepa^ mice (Figure 4a). Obesity is typically characterized by an increase in size and number of adipocytes [42]. The adipocytes in the two WD-fed mice strains were enlarged to a similar degree in comparison to their chow-fed controls. However, in Lsel^−/−^Keap1^flx/flx^ mice, a stronger infiltration of immune cells was visible (Figure 4b). This was supported by flow cytometric analysis. Whilst there was no striking difference in the numbers of CD45^+^ cells/g of adipose tissue between the chow-fed mice strains, the number of these cells was higher in WD-fed Lsel^−/−^Keap1^flx/flx^ mice than in their Lsel^−/−^Keap1^Δhepa^ counterparts (1.7 × 10^6^ ± 1.6 × 10^6^ cells/g fat of Lsel^−/−^Keap1^flx/flx^ mice versus 1.14 × 10^6^ ± 5.94 × 10^5^ cells/g fat of Lsel^−/−^Keap1^Δhepa^ mice). In Lsel^−/−^Keap1^flx/flx^ mice, WD treatment resulted in a significantly increased frequency of CD4^+^ and CD8^+^ T cells, which was not seen in the Lsel^−/−^Keap1^Δhepa^ mice, while there were no significant differences in the frequencies of B cells or any other of the innate immune cells analyzed. (Figure 4c,d).

### 2.5. Nrf2 Activation in Hepatocytes of L-Selectin-Deficient Mice Has Only Minor Effects on WD-Induced Gut Inflammation

**Figure 5:**
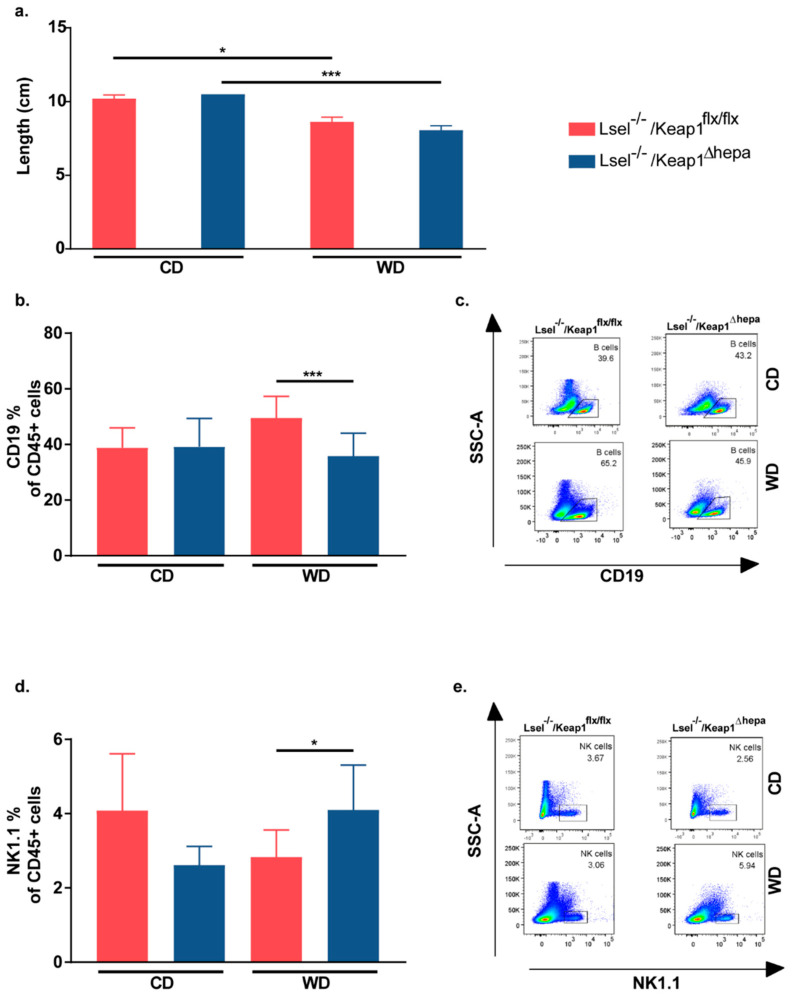
Keap1 inactivation in L-selectin-deficient mice has only minor effects on WD-induced gut changes. Lsel^−/−^Keap1^flx/flx^ mice (shown in red) and Lsel^−/−^Keap1^Δhepa^ mice (shown in blue) were fed for 24 weeks on chow diet (CD) or Western diet (WD). Depicted are the endpoint values of Lsel^−/−^Keap1^flx/flx^ CD-fed (*n* = 6), Lsel^−/−^Keap1^flx/flx^ WD-fed, *n* = 12; Lsel^−/−^Keap^∆hepa^ CD-fed, *n* = 4, and Lsel^−/−^Keap^∆hepa^ WD-fed mice *n* = 12 (**a**) Colon length. (**b**,**d**) Comparative immune cell analysis of colon lamina propria by flow cytometry and (**c**,**e**) corresponding representative FACS dot plots. Representative FACS dot plots illustrating the gating strategy are shown in Supplementary Figure S1. Depicted are the percentages of CD45^+^ cells of (**b**) B cells (CD45^+^CD3^-^CD19^+^) and (**d**) NK cells (CD45^+^NK1.1^+^). Statistical significance was calculated by the one-way ANOVA. Values are represented as either as mean ± SD (**a**,**b**,**d**). * *p* ≤ 0.05, *** *p* ≤ 0.001.

WD-induced gut changes are thought to promote low-grade inflammation and metabolic syndrome. Thus, we next performed gut analysis of Lsel^−/−^Keap1^Δhepa^ and Lsel^−/−^Keap1^flx/flx^ mice. Colon length is known to be negatively associated with inflammation [43]. Indeed, the consequence of feeding both mouse strains with WD was a significant shortening of the colon in comparison to the respective chow-fed controls. At the same time, there was no difference between different mouse strains fed the same diet (Figure 5a). WD-induced low grade inflammation is usually more subtle and does not involve the large inflammatory immune cell infiltrates typically seen in acute infection or inflammatory bowel disease [44]. Accordingly, we did not detect significant WD-induced inflammatory infiltrates and epithelial damage upon histological scoring of the colon and the large intestine in either mouse strain (Supplementary Figure S3). Next, we used flow cytometry to analyze the impact of WD on the adaptive immune cell populations of the gut in more detail. We observed significantly higher frequencies of CD19^+^ B cells in the colonic lamina propria of WD-treated Lsel^−/−^Keap1^flx/flx^ mice compared to similarly treated Lsel^−/−^Keap1^Δhepa^ littermates. In contrast, NK1^+^ NK cells were significantly increased in WD-fed Lsel^−/−^Keap1^Δhepa^ mice (Figure 5b–e). Apart from that, there were no major diet or genotype-induced changes in frequencies of CD4^+^ T cells, CD8^+^ T cells, CD19^+^ B cells, and NK1^+^ NK cells in the lamina propria and the intestinal epithelial fractions of the small and large intestine (data not shown).

### 2.6. Fecal Microbiota Profiles Are Altered by WD-Feeding but Not by Hepatic Nrf2 Activation

**Figure 6:**
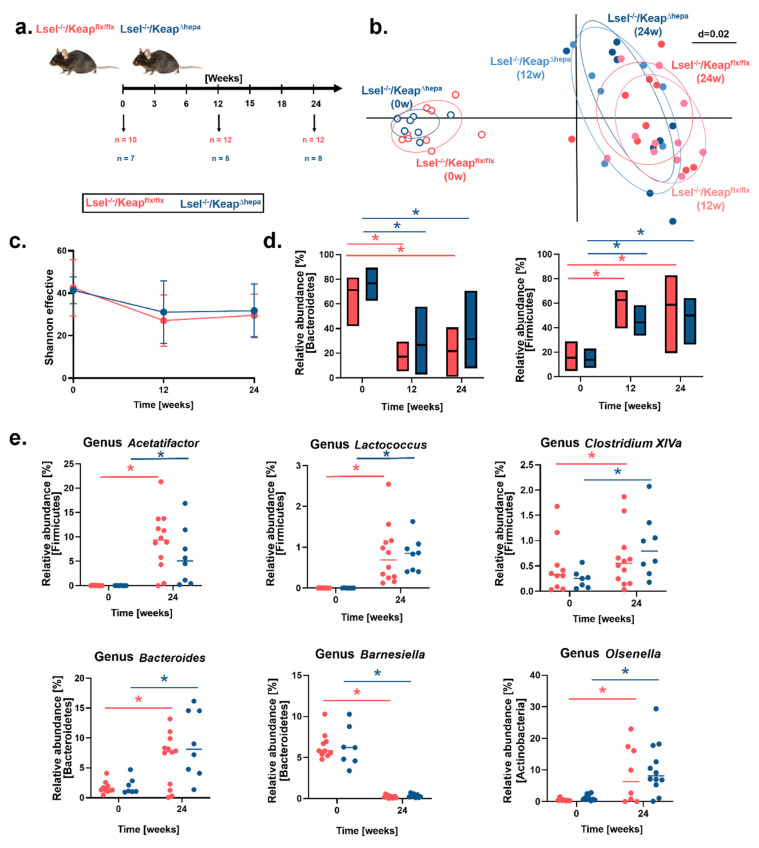
WD feeding causes robust shifts in gut microbiota composition. Lsel^−/−^Keap1^flx/flx^ mice (shown in red) and Lsel^−/−^Keap^∆hepa^ mice (shown in blue) were fed for 24 weeks with Western diet (WD). (**a**) Schematic diagram of the sampling strategy. Fecal samples were collected of Lsel^−/−^Keap1^flx/flx^ and Lsel^−/−^Keap^∆hepa^ littermates at the indicated time points. The number of samples (n) has been demarcated under each point. (**b**) NMDS plot of microbiota profiles based on generalized UniFrac distances from Lsel^−/−^Keap1^flx/flx^ and Lsel^−/−^Keap^∆hepa^ mice at different time points. Individual time points that were considered for calculations have been marked within their respective circles. (**c**) Diversity and composition of the microbiota from different mouse groups. Shown is the Shannon effective count in samples based on time (treatment duration). The diversity of operational taxonomic units (OTUs) within a given sample (alpha-diversity) was calculated in Rhea. (**d**) Taxonomic binning of fecal microbiota of Lsel^−/−^Keap^flx/flx^ and Lsel^−/−^Keap^∆hepa^ mice at the phylum level. (**e**) Relative abundance of various genera in feces of Lsel^−/−^Keap^flx/flx^ and Lsel^−/−^Keap^∆hepa^ mice. In all graphs shown, significance was calculated by non-parametric ANOVA (Kruskal–Wallis Rank Sum Test), * *p* < 0.05.

Obesity and the metabolic syndrome are associated with an altered gut microbiota with decreased bacterial richness. One reason for this could be a reduced bile flow, which is caused by disturbed hepatocyte functions. Bile salts and other products of hepatocytes regulate nutrient uptake, metabolism, microbiota composition, and barrier function in the gut [45]. Amplicon sequencing (16S rRNA gene) was used to investigate whether WD altered the gut microbiota in L-selectin-deficient mice and what influence hepatocytic Nrf2 activation might have. Fecal samples were collected from co-housed WD-fed Lsel^−/−^Keap1^Δhepa^ and Keap1^flx/flx^ littermates at the start (0 weeks, 17 samples), mid (12 weeks, 20 samples), and end (24 weeks, 20 samples) point of feeding (Figure 6a). Across the 57 samples studied, 736,474 high-quality and chimera-checked sequences (12,921 ± 3331 per sample) were analyzed, representing 190 operational taxonomic units (OTUs) (122 ± 17 OTUs per sample). Sequencing depth was evaluated by means of rarefaction curves and, as apparent from the observed plateau in these curves, all samples passed the quality check and were analyzed further (Supplementary Figure S4). Differences in microbiota profiles between the genotypes at the different time points were assessed by non-metric multidimensional scaling (NMDS) visualization of generalized UniFrac distances (Figure 6b). In comparison to the drastic shift in microbiota profiles due to WD feeding, Lsel^−/−^Keap1^Δhepa^ and Keap1^flx/flx^ littermates displayed similar profiles at each time point. Next, alpha-diversity was explored via calculation of the Shannon effective, which revealed no significant difference between the mouse strains (Figure 6c). In addition, the relative abundance of major bacterial phyla (Bacteroidetes, Firmicutes, Actinobacteria, Proteobacteria, Tenericutes, Verrumicrobia) was calculated (Figure 6d and Supplementary Figure S5a). Here again, we detected no differences between Lsel^−/−^Keap1^Δhepa^ and Keap1^flx/flx^ littermates at any time point, but overall, the relative abundance of Bacteroidetes was significantly higher at the starting point, whereas the proportions of Firmicutes were significantly raised after WD feeding. WD feeding increased the ratio of Firmicutes to Bacteriodetes (F/B) in both mouse strains from 0.22 (week 0) to 3.64 (week 12), decreasing to 2.70 (week 24) in Lsel^−/−^ Keap1^flx/flx^ mice, whereas in Lsel^−/−^Keap1^Δhepa^ littermates, the F/B ratio increased from 0.18 (week 0) to 1.66 (week 12) and decreased to 1.59 (week 24) (Supplementary Figure S5b). Although not significant, Actinobacteria and Proteobacteria tended to be more abundant in the WD-fed mice irrespective of the genotype, whereas the relative abundance of Tenericutes decreased upon WD feeding and Verrumicrobia seemed not to be affected (Supplementary Figure S5a). The WD-induced increase in the relative abundance of Firmicutes, which was unaffected by the mouse genotype, was mainly accounted for by an increase in Clostridiales (from 6 to 35% rel. abund.), Lachnospirales (from 4 to 20% rel. abund.), and Erysipelotrichiales (from 4 to 15% rel. abund.). At the genus level, these changes were characterized by increased Acetatifactor, Lactococcus, and Clostridium XIVa, as well as increased unknown members of Lachnospiraceae, Ruminococcaceae, and Erysipelotrichiaceae (Figure 6e and Supplementary Figure S5c). The WD-induced decrease in the relative abundance of Bacteriodetes was mostly accounted for by an unknown genus belonging to Porphyromonadaceae and members of Barnesiella. Interestingly, the relative abundance of Bacteroides increased with WD (Figure 6e and Supplementary Figure S5c). Within the Actinobacteria, an increase in the relative abundance of Olsenella was noticed upon WD feeding (Figure 6e) and within the Proteobacteria, the relative abundance of an unknown genus of the Desulfovibrioaceae was increased (Supplementary Figure S5c).

## 3. Discussion

NAFLD and its subsequent complications are growing global health problems. The disease progression of NAFLD, eventually leading to liver damage, is far from fully characterized. It involves a complex interplay of imbalanced inflammatory cell populations and inflammatory signals derived from liver, adipose tissue, and gut [5]. L-selectin-deficient mice are known to be partially protected from WD-induced NAFLD. By comparing these mice with littermates with an additional hepatic Nrf2 activation in the WD-induced obesity model, we wanted to analyze the effects of simultaneously blocking immune cell infiltration and oxidative stress on NAFLD. We found that in terms of metabolic parameters, additional upregulation of the anti-oxidative stress response further improved NAFLD outcome in terms of metabolic changes in L-selectin-deficient mice. Unexpectedly, it had no additional effects on NAFLD progression and microbiota changes.

We have recently shown that in comparison to wild-type mice, L-selectin-deficient mice exhibited reduced fat accumulation and improved glucose tolerance after WD feeding, compared to wild-type controls [19]. In this study, we identified that this phenotype was further improved by the additional activation of Nrf2 in hepatocytes of Lsel^−/−^Keap1^Δhepa^ mice who display a significant decrease in body weight, eWAT/body weight, and glucose response compared to Keap1^flx/flx^ mice. 

In addition to Nrf2 activation of cytoprotective/antioxidant pathways, there is also evidence that Nrf2 can crosstalk with metabolic pathways. Similar to Lsel^−/−^Keap1^Δhepa^ mice, mice with only hepatocyte-specific Keap1 deletion have been shown to exhibit reduced body weight and peripheral abdominal fat deposition upon WD feeding when compared with similarly treated wild-type mice. This phenotype was accompanied by a comparatively inhibited expression of lipogenic genes [33]. Moreover, mice with hypomorphic Keap1 alleles and resulting Nrf2 activation were partially protected from obesity and more glucose tolerant, which was attributed to a lower expression of gluconeogenic and lipogenic genes mediated by Nrf2-activated adenosine monophosphate (AMP)-activated protein kinase (Ampk) signaling [46]. 

Nrf2 activation in Lsel^−/−^Keap1^Δ^^hepa^ mice resulted in increased hepatic expression of Nrf2 target genes, in particular of GSTM-1 and NQO-1. The antioxidant flavoprotein NQO1 uses (nicotinamide adenine dinucleotide) NADH as electron donor to catalyze the reduction of quinone metabolites, thereby generating increased intracellular levels of NAD^+^. NAD^+^ is a central regulator in cellular energy metabolism. Increased NAD^+^ levels initiate a series of events leading to calorie restriction and elevated mitochondrial respiration. [47,48]. Most interestingly, pharmacological stimulation of NQO1 in mice has been shown to reduce body weight, adipose tissue, related serum parameters, and liver steatosis [49]. Therefore, increased NQO1 levels in Lsel^−/−^Keap1^Δ^^hepa^ mice may be responsible for the observed improvement in metabolic dysfunction. In addition, it has also been shown that GLRX deficiency promotes steatosis and hepatic lipogenesis under certain diets, so that the activation of this gene could also have contributed to the observed beneficial effects [50].

L-selectin-deficient mice exhibited less steatosis and steatohepatitis upon WD feeding [19]. Addition of the hepatocytic Keap1 deletion failed to confer any additional benefits, suggesting the benefits of these two mutations are not cumulative. This was unexpected, as mice with Nrf2 activation have been shown to develop less liver steatosis when compared to wild types [33,46]. In addition, Lsel^−/−^Keap1^Δ^^hepa^ mice exhibited no major alteration in inflammatory gene expression and it was only liver damage, as detected by transaminases and ALP levels, that was slightly improved. This result is in line with our expectations, as the Keap1 deletion alone has not been shown to reduce liver inflammation [33]. Interestingly, in homeostasis, Lsel^−/−^Keap1^Δhe^^pa^ mice exhibited slightly elevated levels of some inflammatory mediators and, although not significant, a trend toward an increased NAS score when compared to their Keap1^flx/flx^ littermates. This result indicates that hepatocyte-specific Keap1 deletion per se is not beneficial under conditions of homeostasis. Nrf2 deletion or suppression was found to be detrimental for liver regeneration [51], increased WAT inflammation [52], and accelerated NASH [53], whereas Nrf2 activation in disease models prevented metabolic dysregulation and decelerated NASH [36,54]. However, Nrf2 activation in leptin-deficient mice elicited conflicting results by worsening steatosis and glucose tolerance [55]. Furthermore, Nrf2 deficiency protected from fibrosis and tumorigenesis in mice with defective hepatic autophagy [56]. A more directed approach would be the specific activation of only one or two Nrf2 target genes such as NQO1, thereby reducing the number of unwanted side effects.

Excess adipose tissue increases the secretion of a range of factors such as adipokines, cytokines, and fatty acids, predisposing individuals to the development of NAFLD. Additional hepatic Nrf2 activation improved the metabolic phenotype of WD-fed L-selectin-deficient mice, as exhibited by reduced visceral body fat with fewer immune cell infiltrations. Concerning the gut, both mouse mutants displayed a significant shortening of the colon upon WD feeding. This could indicate WD-induced low-grade inflammation, although the frequencies of CD19^+^ B cells in the lamina propria of Lsel^−/−^Keap1^Δ^^hepa^ were significantly reduced, and we did not observe any noticeable histological changes. 

Previous research has shown that transferring the microbiota from obese to germ-free mice led to increased weight and fat gain, highlighting the impact of the gut microbiota [57,58]. This effect is further influenced by diet, which impacts both the host and the microbiota [59]. Due to this, the WD has become a standard model for reproducibly altering the gut microbiota, which in turn influences the host. In fecal samples of Lsel^−/−^Keap1^Δhepa^ mice and Keap1^flx/flx^ littermates fed a WD, two distinct microbial community clusters were identified, corresponding to the two dietary conditions. WD feeding dramatically altered microbiota profiles after 12 weeks with no further alterations at 24 weeks. However, no genotype-dependent clustering was observed. The WD diet significantly altered the taxonomic profile at both phyla and genus level, with reduced bacterial richness and an increased Firmicutes/Bacteroidetes ratio. According to Xiao et al., Clostridia are enriched upon WD feeding, thereby accelerating bile acid 7-alpha dehydroxylation [60]. Consistent with that report, our study also shows a WD-induced relative increase in Clostridiales and Lachnospirales with increased relative abundance of *Clostridium XIVa* and *Acetatifactor*, respectively. This also corresponds to reports of increased abundance of *Clostridium* in obese mice and in a Westernized humanized mouse model [61] and higher abundance of *Acetatifactor* upon high-fat diet feeding [62]. In addition, *Erysipelotrichiaceae*, also members of the Firmicutes, were found to be strongly increased. Turnbaugh et al. [61] and Fleissner et al. [63] previously reported increased representation of *Erysipelotrichiaceae* in WD-associated humanized mouse microbiota and WD-fed conventional mice, respectively. Another genus belonging to Firmicutes that is associated with diet-induced obesity, in mice, is *Lactococcus* [64]. Our observation of a relative increase in *Lactococcus* after WD feeding supports this. Within the Bacteroidetes phylum, WD caused a drastic decrease in the *Porphyromonadaceae*, which represented the most abundant family detected. This corroborates former studies [62]. In particular, the abundance of *Barnesiella*, a bacterium associated with ‘lean microbiota’ and negatively associated with metabolic syndrome phenotype [62,65,66], was strongly reduced. In contrast, the relative abundance of *Bacteroides* was increased by WD feeding. *Bacteroides* were shown to be enriched in Type 2 diabetes in humans consuming a high-fat diet, and they are associated with long-term consumption of a fat-enriched diet [67,68,69]. They might indicate potential inflammation, as members of this genus have previously been associated with colitis [70]. With regard to Proteobacteria, WD caused a significant increase in an unknown genus of *Desulfovibrionaceae*. This result is in line with studies showing a positive correlation between fat intake and Proteobacteria [71,72]. According to Shin et al., abnormal growth of Proteobacteria may reflect an imbalance in the gut microbial population and be a potential marker of disease risk [73]. The phylum Actinobacteria was dominated by the genus *Olsenella*, which was often strongly increased in WD-treated mice. This was unexpected, as *Olsenella* is more abundant in lean people compared to obese people and was reported to decrease in mice subjected to high-fat feeding [65,74,75,76]. In addition, the relative abundance of *Ruminococcaceae* (phylum Firmicutes) was found to be increased by WD. These bacteria, such as *Olsenella*, can produce short chain fatty acids, and they are thought to protect the gut barrier and provide energy for intestinal cells. Overall, even though WD-induced changes in the fecal microbiota were observed in our study, the lack of difference between the two mouse strains (*Lsel^−/−^Keap1^flx/flx^* vs. *Lsel^−/−^Keap^∆hepa^*) suggests that Nrf2 activation in hepatocytes has no relevance for the composition and constitution of the gut microbiota under these conditions.

In conclusion, we have demonstrated that an upregulation of the anti-oxidative stress response in hepatocytes improves the metabolism of L-selectin-deficient mice under conditions of WD feeding, but it confers no benefit in relation to homeostasis. Furthermore, it does not prevent NAFLD progression and associated changes in the microbiome. These findings extend our understanding of the processes underlying NAFLD development, which will be important for the development of new therapies. 

## 4. Materials and Methods 

### 4.1. Housing, Generation of Mice, and Dietary Treatments 

Animals were housed in the animal facility of the University Hospital RWTH Aachen with 12 h light/dark cycles and water and food available ad libitum.

Hepatocyte-specific Keap1 deletion in L-selectin-deficient mice was achieved by crossing floxed-Keap1 mice, expressing Cre recombinase heterozygote under the regulation of the Albumin gene (Alb-Cre mice) [31,33], with L-selectin-deficient mice [77], all on a C57BL76J background. The resulting L-selectin-deficient Alb-cre negative Keap1fl/fl mice (Lsel^−/−^Keap1^flx/flx^) and Alb-cre positive littermates with hepatocyte-specific conditional Keap1 deletion (Lsel^−/−^Keap1^Δhepa^) were used as experimental animals. 

Western diet (WD) treatments were performed with 9–15-week-old male mice weighing at least 25 g. Mice were fed first chow (9 kcal % fat, 24 kcal % protein, 67 kcal % carbohydrates) (ssniff, Soest, Germany, rat/mouse–maintenance) or WD (40 kcal % fat (vegetable fats, 20 kcal % fructose, 2% cholesterol) (Brogaarden, Lynge, Denmark; cat. no. D09100301) for 24 weeks. Food intake and body weights were measured weekly. All experiments were repeated in two independent experimental setups. 

### 4.2. Glucose Tolerance Test (GTT) 

Mice were fasted for 6 h and blood glucose was measured using an Accu-Check^®^ Aviva meter (Roche, Basel, Switzerland), via one drop of blood taken from the animal’s tail, every 15 min for 2 h following intraperitoneal administration of 2 g/kg glucose.

### 4.3. Serum and Liver Biochemical Measurements

Serum aspartate aminotransferase (AST), serum alanine aminotransferase (ALT), alkaline phosphatase (ALP), glucose, triglyceride, and cholesterol levels in serum were measured by the Central Laboratory Facility of the University Hospital, RWTH Aachen. For the determination of the intrahepatic triglyceride (TG) concentration, 20 mg liver was homogenized in 1 mL of a homogenization buffer (10 mM Tris, 2 mM EDTA, 0.25 M sucrose, pH 7.5), and the assay was performed in accordance with the manufacturer’s instructions of the Instruchemie LiquiColor mono Kit (Instruchemie, Delfzijl, the Netherlands). For intrahepatic free fatty acid (FFA) quantification, the total amount of lipids within 20 mg snap-frozen liver tissue were extracted with chloroform-Triton X-100. The concentration of FFA was measured with the FFA quantification kit (Abcam, Cambridge, UK; cat. no ab65345) in accordance with the manufacturer’s instructions.

### 4.4. Histological Stainings

Following fixation with 4% formalin/PBS, livers, intestines, and adipose tissue were embedded in paraffin. Then, 3 μm paraffin sections were serially cut, mounted onto glass slides, deparaffinized, and stained with H&E. Histopathological scoring of sections from liver, colon, and small intestine and their validation was performed blinded via an NAFLD activity score (NAS), a colon score, and a score for the small intestine, as described previously [78,79,80].

For immunofluorescence staining of hepatocytes, hepatocytes were allowed to attach to collagen-coated cover slips and were then fixed in 4% paraformaldehyde, permeabilized in 0.2% Triton X-100, blocked with 2% goat serum, and incubated with a rabbit-anti-Nrf2 antibody for 1 h at room temperature. Then, they were treated with a secondary Cy3 labeled goat-anti-rabbit IgG for 1 h in the dark at room temperature (the antibodies utilized are listed in Table 1 below). Nuclei were stained for 2 min with DAPI. Images were acquired using an Axioplan2 fluorescence microscope (Carl Zeiss Microscopy, Oberkochen, Germany) and Zen lite software (Carl Zeiss Microscopy, Oberkochen, Germany).

### 4.5. Isolation of Cells and Flow Cytometry

For flow cytometric analysis of intraheptatic leukocytes, livers were perfused with phosphate-buffered saline (PBS), minced with scissors, and digested for 45 min in RPMI medium containing 10% fetal calf serum (FCS), 1% penicillin/streptomycin, 1.25 mg/mL collagenase D, and 3 µL/mL (stock: 10 mg/mL) DNAse 1. After stopping the enzyme activity with 2 mM EDTA, the digestion mixture was passed through 40 µm cell strainers. After centrifugation for 10 min at 400× *g*, the suspension was subjected to gradient centrifugation using 35% percoll at 750× *g* for 20 min to remove debris. The cell sediment was subjected to red cell lysis using Pharmlyse (BD) and after washing was ready for staining. Hepatocytes, adipose tissue, gut IELs, and LPLs were prepared as described previously [81,82,83,84]. Single cell suspensions were stained directly using combinations of the monoclonal antibodies listed in Table 1. 

Intracellular Nrf2 staining was performed with an intracellular Foxp3 staining buffer set (eBioscience, Frankfurt, Germany). Hepatocytes from Nrf2^−/−^ mice [85] were used as negative control in addition to a florescence minus one (FMO) control. 

To analyze the net intracellular generation of ROS, the cellular fluorescence intensity was measured after 60 min incubation of hepatocyte suspensions with ROS Assay Stain in accordance with the manufacturer’s instructions of the Total Reactive Oxygen Species (ROS) Assay Kit (Invitrogen, Waltham, MA, USA). Anti-CD45 and anti-CD146 antibodies were added for the last 15 min of the incubation. Cells that were negative for CD146 and CD45 were analyzed for Nrf2 expression. Similarly treated cells incubated at 4 °C served as negative control. 

All flow cytometric measurements were performed on a Canto-II cytometer (BD Biosciences). Data were analyzed by FlowJo 8.7.2 and 10.2 software (Tree Star, Ashland, OR, USA).

### 4.6. Gene Expression Analysis by Real-Time PCR

Total RNA isolations from the liver and complementary DNA (cDNA) synthesis were performed as described previously [79]. Real-time polymerase chain reactions (RT-PCR) were performed in duplicate in a total volume of 20 µL, on a 7300 RT-PCR system with 7000 System SDS Software Version 1.2.3 (Applied Bioscience, Darmstadt, Germany) using the quantitative (q)PCR Master Mix for SYBR Green I (Eurogentec, Cologne, Germany). Primer sequences are listed in Table 2. Glyceraldehyde 3-phosphate dehydrogenase (*GAPDH* was used as endogenous control for normalization).

### 4.7. DNA Isolation from Feces, 16S rRNA Gene Amplicon Sequencing, and Data Analysis

Metagenomic DNA was extracted from frozen fecal pellets by bacterial disruption using zirconia beads (Biospec) and a FastPrep 24 device (MP Biomedical, USA) at 6 m/s for 20 s. Homogenates were incubated at 80 °C for 10 min, centrifuged at 17000×g for 1 min, and supernatant was used for DNA isolation using the QIAamp fast DNA stool kit (Qiagen, Cat. No. 51604) according to the supplier’s instruction. The V3/V4 region of 16S rRNA genes was amplified (25 cycles) from 24 ng of metagenomic DNA using primer 341F and 785R in a two-step procedure to limit amplification bias [86,87]. Libraries were double-barcoded (8-nt index on each of the forward and reverse 2nd-step primer) [88,89]. Amplicons were purified using the AMPure XP system (Beckmann Coulter Biomedical GmbH), pooled in an equimolar amount with addition of 25% (*v*/*v*) PhiX library, and sequenced in paired-end modus (PE275) using a MiSeq system (Illumina).

Then, 16s rRNA amplicon sequencing was done at the Core Facility Microbiome/NGS at the Technical University of Munich. Data processing was done using the IMNGS platform [90], applying the UPARSE analysis pipeline [91] with the following settings: Number of allowed mismatches in the barcode: 1; Minimal fastq quality score for trimming of unpaired reads: 20; Maximal rate of expected errors in paired sequences: 2; Min relative abundance of operational taxonomic units (OTUs) cutoff (0–1): 0.25%. The data were submitted to the Sequence Read Archive and are available under the accession number PRJNA714661. 

### 4.8. Statistical Analysis of Microbiota Sequencing

Statistical analysis of data from mouse experiments was performed with GraphPad Prism software (version 7; GraphPad, La Jolla, CA, USA). Data are presented as mean ± Standard Deviation (SD). Significance values were calculated using the Student’s *t-*test when comparing two groups or one-way analysis of variance (ANOVA) and Sidak or Tukey post-test. Values of *p* < 0.05 were considered significant (* *p* < 0.05, ** *p* < 0.01, *** *p* < 0.001, and **** *p* ≤ 0.0001). 

Statistical analysis of data from microbiota sequencing were performed using Rhea in the R programming environment [92]. Alpha- and beta-diversity were calculated from normalized data using generalized UniFrac distances in the latter case. Visualization of the multidimensional distance matrix was achieved through either Multi-Dimensional Scaling (MDS) or its non-metric counterpart (NMDS).

## Figures and Tables

**Table 1:** Antibodies used in this study.

Antibody	Channel	Manufacturer	Clone	Dilution
NK 1.1	AF 488	Biolegend	PK136	1:400
CD4	PE	BD biosciences	GK1.5	1:400
CD45	APC-Cy7	BD biosciences	30-F11	1:400
CD19	eFluor 450	eBioscience	1D3	1:200
CD8a	Amcyan	Biolegend	53-6.7	1:400
CD3e	PerCp-Cy5.5	eBioscience	145-2C11	1:100
F4/80	eFluor 450	Serotec	CI:A3-1	1:200
CD11b	Amcyan	BD Biosciences	M1/70	1:400
Ly6G	FITC	BD Biosciences	1A8	1:400
Calibrite counting beads	APC	BD Biosciences		1 million beads/mL PBS
CD146	APC	Miltenyi	ME-9F1	1:400
Nrf2		GeneTex	N2C2	1:400
Goat anti-rabbit IgG	Cy3	Invitrogen		1:500
Goat anti-rabbit IgG	AF 633	Invitrogen		1:500

**Table 2:** Primers used in this study.

Primer	Forward	Reverse
GAPDH	ACCTGCCAAGTATGATGACATCA	GGTCCTCAGTGTAGCCCAAGAT
NQO-1	AGAGAGTGCTCGTAGCAGGAT	CTACCCCCAGTGGTGATAGAAA
TNF-α	CATCTTCTCAAAATTCGAGTGACAA	TGGGAGTAGACAAGGTACAACCC
IFN-γ	GAGGTCAACAACCCACAGGTC	CGAATCAGCAGCGACTCCT
IL-6	CTGGAGTCACAGAAGGAGTGG	GGTTTGCCGAGTAGATCTCAA
IL-1β	GATCCCAAGGCAATACCCAAA	GGGGAACTCTGCAGACTCAA
Foxp3	GGCAAATGGAGTCTGCAAGTG	CAGGAGATGATCTGCTTGGCA
GLRX	TTATAAAAGGGGTGGCAGGCAG	GTTAAGCTTCTCGGCCCCAT
GSTM1	CCTGGATGGAGAGACAGAGG	GACCTTGTCCCCTGCAAA
GCLC	GGCACAAGGACGTTCTCAAGT	CAAAGGGTAGGATGGTTTGGG

## Data Availability

The microbiota sequencing data presented in this study are openly available under the accession number PRJNA714661.

## Supplementary Materials

Supplementary materials can be found at https://www.mdpi.com/article/10.3390/ijms22147314/s1. Figure S1: Flow cytometric analysis of intracellular ROS in hepatocytes. Figure S2: Immune cell subset gating by multiparameter flow cytometry. Figure S3: WD-feeding does not cause significant histological gut changes. Figure S4: Rarefaction curves depicting sequencing depth. Figure S5: WD-feeding causes robust shifts in gut microbiota composition.

## Abbreviations

AF488	Alexa Fluor 488
APC-Cy7	Allophycocyanin-cyanine 7
CD	Chow diet
GAPDH	Glyceraldehyde 3-phosphate dehydrogenase
GCLM	Glutamate-cysteine ligase modifier
GLRX	Glutaredoxin
GSTM1	Glutathion S-transferase1
HO-1	Heme oxygenase-1
IFN-γ	Interferon γ
IL-6	Interleukin-6
NAFLD	Non-alcoholic fatty liver disease
NASH	Non-alcoholic steatohepatitis
NK	Natural killer
NQO-1	NAD(P)H quinone dehydrogenase 1
PE	Phycoerythrin
PerCP-Cy5.5	Peridinin chlorophyll protein-cyanine5.5
ROS	Reactive oxygen species
TNF-α	Tumor necrosis factor- α
TRX	Thioredoixin
WD	Western diet
