# Supplementary material for: Upregulation of Anti-Oxidative Stress Response Improves Metabolic Changes in L-Selectin-Deficient Mice but Does Not Prevent NAFLD Progression or Fecal Microbiota Shifts
Source: Int J Mol Sci. 2021 Jul 7;22(14):7314. doi: 10.3390/ijms22147314 (PMC8306675; doi:10.3390/ijms22147314)
Supplement: Supplementary file 1 [file ijms-22-07314-s001.zip › ijms-1231479-supplementary.pdf]

International Journal of
Molecular Sciences

MDPI

Supplementary Materials

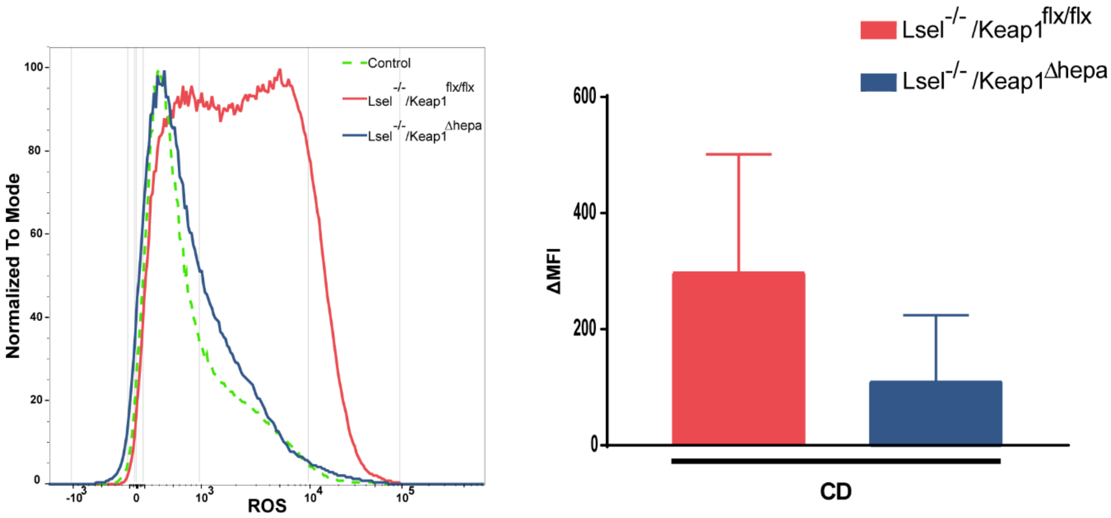

Figure S1. Flow cytometric analysis of intracellular ROS in hepatocytes of Lsel-/-Keap1$^{flx/flx}$ mice and Lsel-/-Keap$^{\Delta hepa}$ mice, showing representative histograms (left side) and the quantification of the ΔMFI (mean fluorescence intensity) (right side) as difference of geometric mean of the measured samples and the respective negative controls (similar sample incubated at 4°C) of hepatocytes from Lsel-/-Keap1$^{flx/flx}$ mice (n=7) and Lsel-/-Keap$^{\Delta hepa}$ mice (n=7). Significance was calculated by non-parametric T test (Mann-Whitney Test), p=0.54.

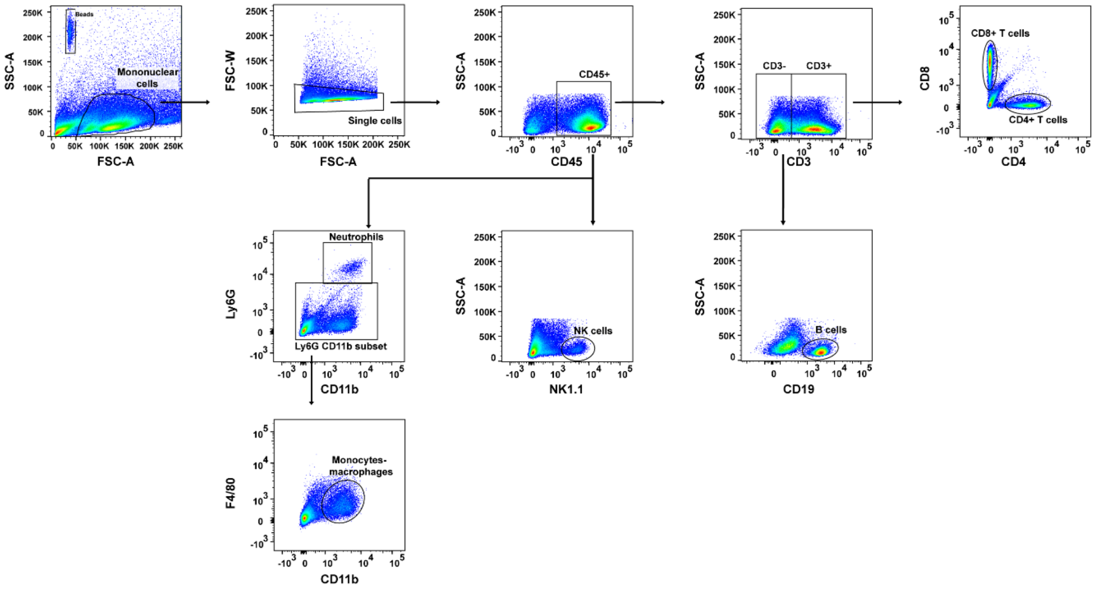

Figure S2. Immune cell subset gating by multiparameter flow cytometry (liver cells, representative of all organ fractions). The analysis included: B cells ($CD45^+CD3^-CD19^+$), $CD4^+$ T cells ($CD45^+CD3^+CD4^+$), $CD8^+$ T cells ($CD45^+CD3^+CD8^+$), natural killer (NK) cells ($CD45^+NK1.1^+$), monocytes/macrophages (Mo-MF)($CD45^+CD11b^+Ly6G^-F4/80^{+/-}$), neutrophils ($CD45^+CD11b^+Ly6G^+$).

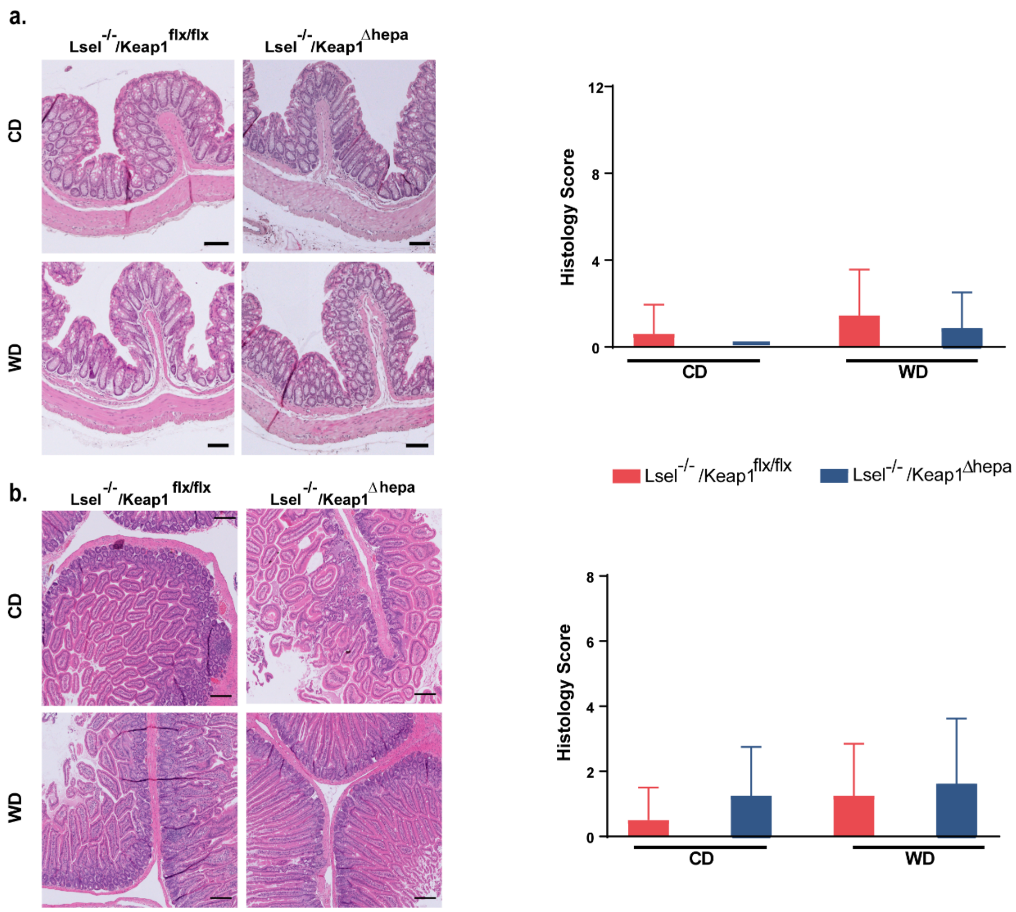

Figure S3. WD-feeding does not cause significant histological gut changes. $Lsel^{-/-}Keap1^{flx/flx}$ mice (shown in red) and $Lsel^{-/-}Keap1^{\Delta hepa}$ mice (shown in blue) were fed for 24 weeks on chow diet (CD) or western diet (WD). **(a)** Representative images of H&E stained colon sections of the indicated mouse strains (original magnification X 10, scale bar = 200 µm) and results of histological scoring ($Lsel^{-/-}Keap1^{flx/flx}$ CD-fed (n=5), $Lsel^{-/-}Keap1^{flx/flx}$ WD-fed (n=11), $Lsel^{-/-}Keap1^{\Delta\ hepa}$ CD-fed (n=4), $Lsel^{-/-}Keap1^{\Delta hepa}$ WD-fed (n=8)). **(b)** Representative images of H&E stained sections of the small intestine of the indicated mouse strains (original magnification X 20, scale bar = 200 µm and results of histological scoring ($Lsel^{-/-}Keap1^{flx/flx}$ CD-fed (n=4), $Lsel^{-/-}Keap1^{flx/flx}$ WD-fed (n=11), $Lsel^{-/-}Keap1^{\Delta\ hepa}$ CD-fed (n=3), $Lsel^{-/-}Keap1^{\Delta hepa}$ WD-fed (n=8)). Statistical significance was calculated by one-way ANOVA.

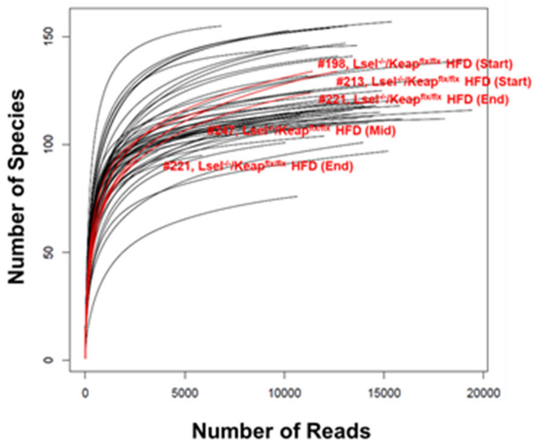

Figure S4. Rarefaction curves depicting sequencing depth. The number of observed species in each sample was plotted against the number of reads acquired, depicting a plateau region. The curve was generated using Rhea [67] by standard normalized counts through simple division by their sample size and then multiplication by the size of the smaller sample. The five samples with the least sequencing reads are shown in red.

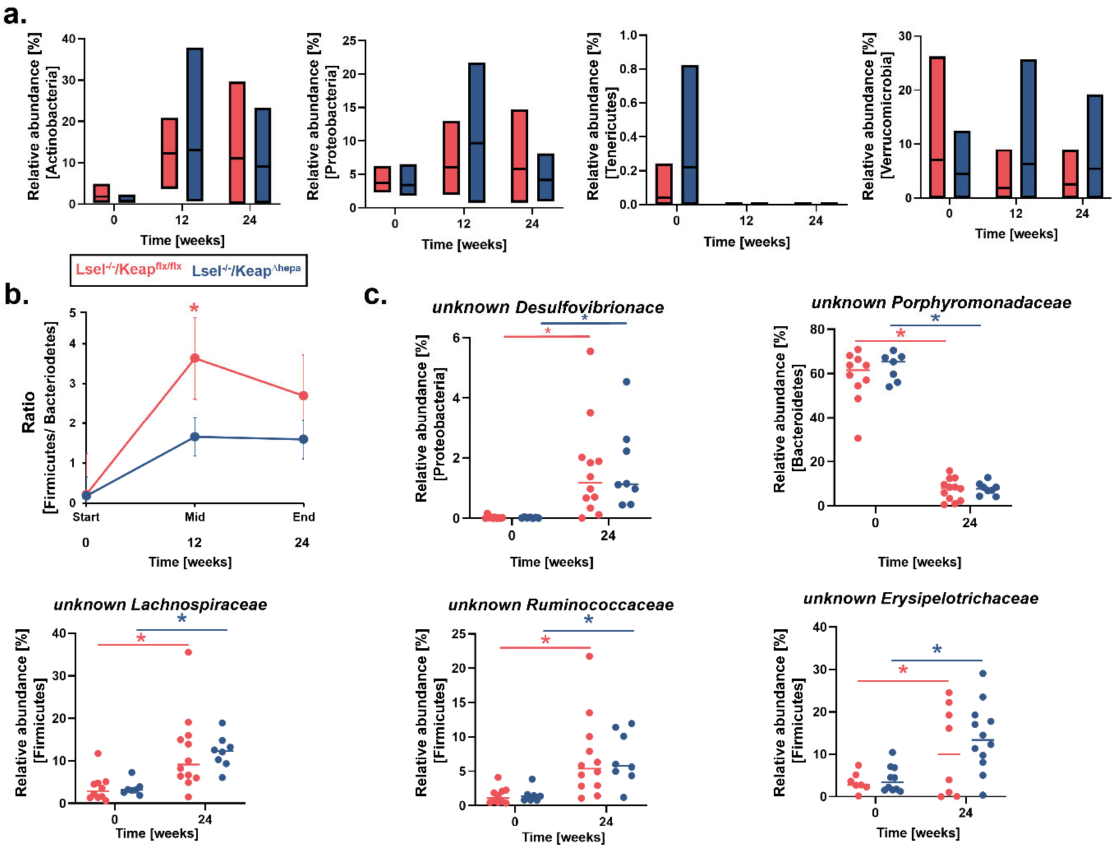

Figure S5. WD-feeding causes robust shifts in gut microbiota composition. Lsel-/-Keap1$^{flx/flx}$ mice (shown in red) and Lsel-/-Keap$^{\Delta hepa}$ mice (shown in blue) were fed for 24 weeks with western diet (WD). Fecal samples were collected at the indicated time points. **(a)** Taxonomic binning of fecal microbiota of Lsel-/-Keap1$^{flx/flx}$ mice and Lsel-/-Keap$^{\Delta hepa}$ mice at the phylum level. **(b)** Firmicutes to Bacteroidetes ratio in feces of Lsel-/-Keap$^{flx/flx}$ and Lsel-/-Keap$^{\Delta hepa}$ mice at different time points. **(c)** Relative abundance of various unknown genera in feces of Lsel-/-Keap$^{flx/flx}$ and Lsel-/-Keap$^{\Delta hepa}$ mice. In all graphs shown, significance was calculated by non-parametric ANOVA (Kruskal-Wallis Rank Sum Test), *$p<0.05$.
